# Burden of neurological diseases in the US revealed by web searches

**DOI:** 10.1371/journal.pone.0178019

**Published:** 2017-05-22

**Authors:** Ricardo Baeza-Yates, Puneet Mohan Sangal, Pablo Villoslada

**Affiliations:** 1 University Pompeu Fabra, Barcelona, Spain; 2 Amazon, Seattle, Washington, United States of America; 3 University of California of San Francisco, San Francisco, United States of America; 4 Institut d’Investigacions Biomediques August Pi Sunyer (IDIBAPS), University of Barcelona, Barcelona, Spain; University of Toronto, CANADA

## Abstract

**Background:**

Analyzing the disease-related web searches of Internet users provides insight into the interests of the general population as well as the healthcare industry, which can be used to shape health care policies.

**Methods:**

We analyzed the searches related to neurological diseases and drugs used in neurology using the most popular search engines in the US, Google and Bing/Yahoo.

**Results:**

We found that the most frequently searched diseases were common diseases such as dementia or Attention Deficit/Hyperactivity Disorder (ADHD), as well as medium frequency diseases with high social impact such as Parkinson’s disease, MS and ALS. The most frequently searched CNS drugs were generic drugs used for pain, followed by sleep disorders, dementia, ADHD, stroke and Parkinson’s disease. Regarding the interests of the healthcare industry, ADHD, Alzheimer’s disease, MS, ALS, meningitis, and hypersomnia received the higher advertising bids for neurological diseases, while painkillers and drugs for neuropathic pain, drugs for dementia or insomnia, and triptans had the highest advertising bidding prices.

**Conclusions:**

Web searches reflect the interest of people and the healthcare industry, and are based either on the frequency or social impact of the disease.

## Introduction

The burden of neurological diseases is prominent around the world, contributing to 2–6% in the World Health Organization’s analysis “Global Burden of Diseases 2010”[1]. Diseases associated with aging, such as Alzheimer’s disease and stroke, are the leading cause of disability and death in developed countries, whereas in developing countries, brain injury and infections are predominant.

An approach to get additional information directly from the general population is analyzing Web searches, known as the infodemiology approach[2]. Even if such information is not structured or systematic, it provides the power of big data for identifying frequencies and trends on diseases from a user / population perspective (e.g. flu outbreaks[3], adverse events due to drug usage[4], or predicting hospital visits [5]). Moreover, patients with neurological diseases are some of the most active on the Web and many are digital health users[6–14]. In parallel, this approach can reveal the interests of the healthcare industry in a given health area by providing information about web search advertising.

In this study, we aimed to analyze the searches related to neurological diseases and drugs for neurological diseases in the US. We enhance previous web search methodology with analysis of bid prices in web search advertising, allowing us to infer the interests of the healthcare industry.

## Methods

### Web searches

Considering differences in the use of search engines, languages and organization of health systems between countries, we only analyzed searches done in English from the US, using aggregated data for 2015. By focusing in only in the US we were able to obtain more homogenous results at the prize of losing generalization to other Western countries and assuming the bias to higher-educated population. All data used in this study was obtained from public advertising interfaces (APIs) available in the two major search engines, Google and Bing/Yahoo. No user identification procedure was performed at any time. Therefore, informed consent was not requested from users as we used aggregated results. For the same reason, we could not do session analysis, in which we would track the sequence of requests of each user (e.g. first searching a symptom such as “weakness”, then for an illness such as “multiple sclerosis”, then a drug such as “interferon beta” or an indication of disability such as “wheelchair”). For this reason, we focused in more precise terminology such as disease diagnosis and did not performed more general, common or vague terms such as “pain”, which prevented the analysis by symptoms. The ethical committee of Hospital Clinic of Barcelona, Spain, approved this study.

### Databases

We used the list of diseases from the National Institute of Neurological Disorders and Stroke (http://www.ninds.nih.gov/disorders), which provided a list of 448 neurological diseases and enriched the list with its common disease abbreviations.

In addition, we created the list of FDA approved drugs for neurological diseases (including pain) from Centerwatch (https://www.centerwatch.com) and Medilexicom (http://www.medilexicon.com) including the generic and brand name. Because some commonly used drugs in Neurology are old (e.g. phenobarbital) or may have not been approved for these indications (off label use), we enriched the list of drugs with the list of drugs reimbursed by Medicare for neurological diseases as described in[15], leading a list of 212 drugs (see S1 File for the full list of term searches used).

### Analysis

We evaluated people’s interest in brain diseases by using the volume of search terms related to neurology, either disease diagnosis or CNS drugs. That is, we considered the frequency or volume of a query (or group of queries that have the same meaning) as a good proxy for the interest of people. Similarly, we used the real-time bids for web queries by advertisers to evaluate the interests of the healthcare industry. In search advertising, a second price auction is performed for each query, where for each click the advertiser pays what the next advertiser is willing to pay. Therefore, the bid value is a good proxy for the interest of the industry (advertiser) for a given query. Hence, the total value of the market for that query, *spend*, is proportional to the query volume multiplied by the bid. This market value can be estimated from different search engines and will give different results due to their different number of users, the number of advertisers, and their technology (e.g. how the bid is suggested to a new advertiser; or how polysemy of terms is handled). For our analysis, we use the standard advertising APIs of Google and Bing/Yahoo restricted to the US market, as they are the major search engines nowadays. For the case of Google, we also include a *competition* factor that estimates the competitiveness for the market of each query, or in other words the number of companies interested in a given term (https://support.google.com/adwords/answer/3022575?hl=en).

## Results

### Frequency of neurological diseases in web searches

**Fig 1:**
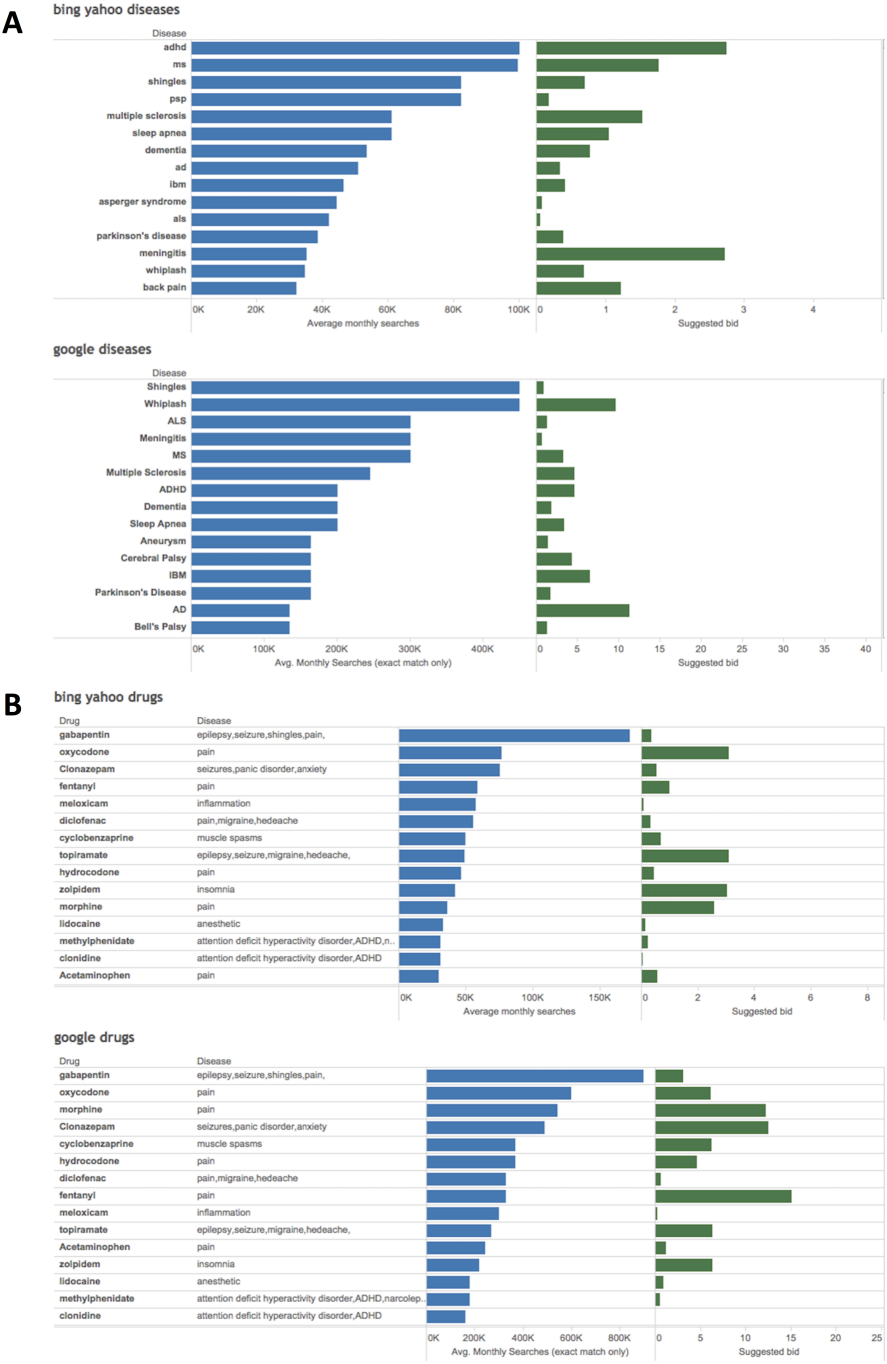
Average monthly searches and bid price of neurological diseases and CNS drugs. A) Figure shows the top 15 diseases (blue bars) and its suggested ad bid (green bars, in USD) searched in the Bing/Yahoo and Google search engines. Full names and well accepted acronyms were used for the analysis. Abbreviations: ADHD: Attention-Deficit/Hyperactivity Disorder; MS: Multiple Sclerosis; PSP: Progressive Supranuclear Palsy; AD: Alzheimer Disease; IBM: Inclusion-Body Myositis; ALS: Amyotrophic Lateral Sclerosis. B) Figure shows the top 15 drugs with their indication (blue bars) and suggested ad bid (green bars, in USD) searched in the Bing/Yahoo and Google search engines.

We evaluated the interest of people in neurological diseases by using the volume of search terms related to neurological diseases (full name or common abbreviations) in the two most important search engines Google and Bing/Yahoo (S1 File). We found that the most searched neurological diseases (Fig 1A) were highly prevalent diseases such as Dementia or Attention Deficit/Hyperactivity Disorder (ADHD), as well as medium frequency diseases with high social impact such as Parkinson disease (PD), Multiple Sclerosis (MS) or Amyotrophic Lateral Sclerosis (ALS) (S2 File). Surprisingly, the two most prevalent neurological diseases, headache and stroke, were not ranked in the top 5 or 10, but they were in the top 30.

Moreover, highly ranked searches included polysemic terms such as “whiplash” or “shingles”. Both engines handle polysemic terms in a different way, which can explain some differences in the frequency of searches. In our queries, we included the name of the disease as well as common disease abbreviations. However, abbreviations can refer to different terms. Nevertheless, the effects of such abbreviations were not significant because they did not modify the ranking of a given condition, with three exceptions: IBM (Inflammatory Body Myositis vs IBM corporation), PSP (Progressive Supranuclear Palsy vs PlayStation Portable) and TM (Transverse Myelitis vs Trademark) (S2 File).

Second, we analyzed the bids for queries by advertisers to evaluate the interest of the healthcare industry in neurological diseases (S2 File). Although the cost of each ad varies among search engines, the bid price is not related to frequency of the disease nor based solely on the frequency of the searches, but also reflects market value in the health sector[16]. Neurological diseases ranking higher in the bids included ADHD, PD, Alzheimer’s disease, MS and meningitis (Fig 1A).

### Frequency of CNS drugs searches

We evaluated the searches for drugs for neurological indications (S2 File). The most commonly searched drugs were generic drugs used for pain, followed by sleep disorders, dementia, ADHD, stroke, or PD (Fig 1B). Moreover, drugs that were approved more recently also ranked high considering their prescription rates compared to those of the massively used drugs described above, such as the case of immunomodulatory drugs for MS, and several anti-epileptics and drugs for PD (S3 File). Regarding the bids for queries by advertisers for CNS drugs, Over The Counter (OTC) drugs including painkillers, drugs for headache, epilepsy and insomnia ranked the highest (Fig 1B).

### Neurology’s market analysis based in web searches

**Fig 2:**
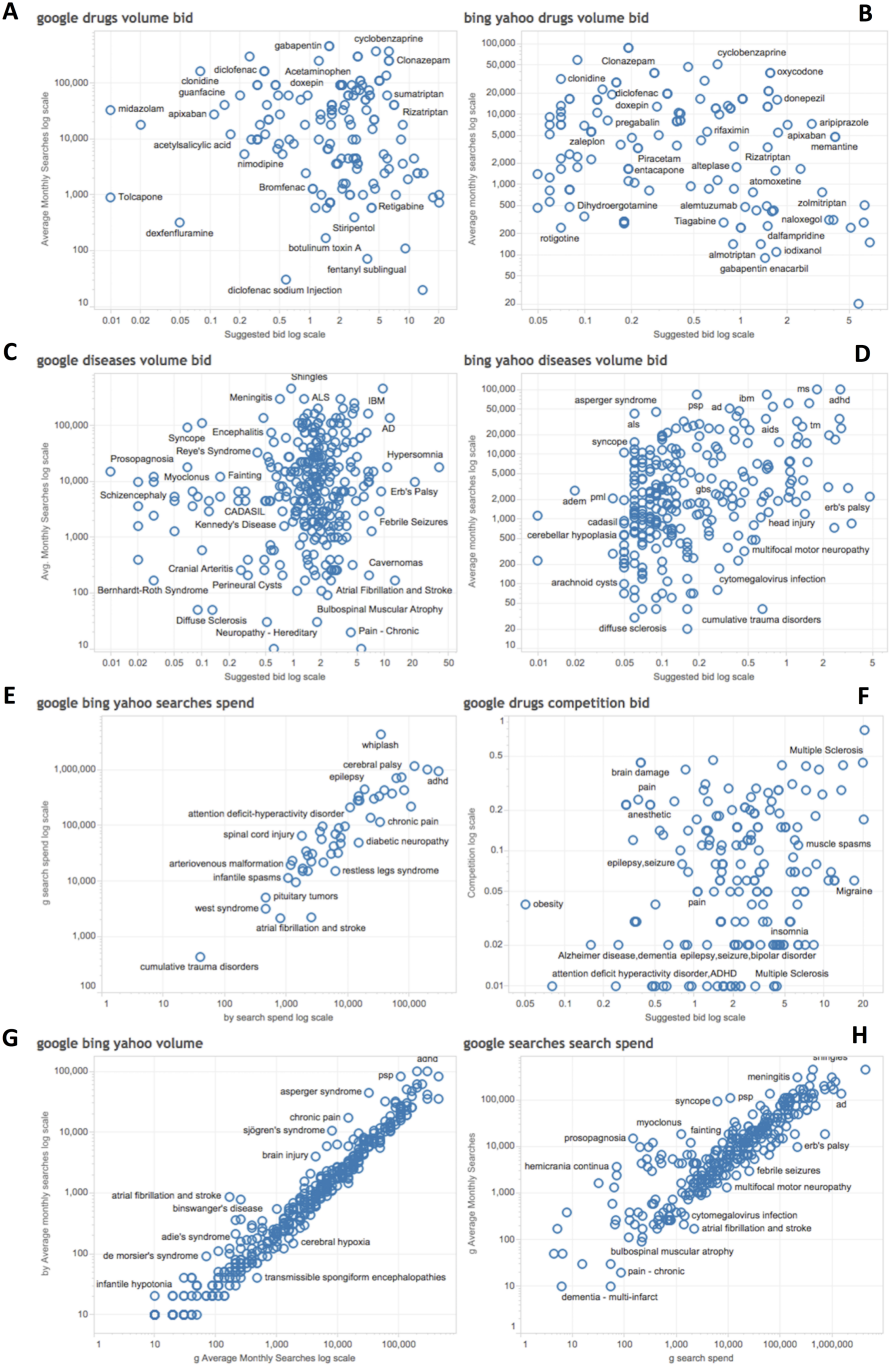
Relationship of query volume and advertising expenditure (ad bid) for CNS drugs and diseases searches. A-D) show the average monthly searches (Y axis) for CNS drugs or diseases and the suggested ad bid (X axis, in log scale) for either drugs or diseases for searches in Google or Bing/Yahoo. In each graph, queries that are in the top represent the people’s interest (searches) while the queries that are in the right side represent the health industry’s interest (suggested ad bid). E-H) show data for Google (*g*) or Bing/Yahoo (*b*) in log scale for searches of neurological diseases. Comparisons are done using the total search spend (E), the Google competition against bid (see Methods for definition) (F), the average monthly searches for Google and Bing/Yahoo (G), and the average monthly searches against the advertising spend for Google (H).

We analyzed the relation between query volume and query bid (value per click) for drugs and diseases for both search engines (Fig 2A–2D). ADHD, Alzheimer disease, MS, ALS, meningitis, and hypersomnia ranked high in both dimensions. Regarding CNS drugs, painkillers, triptans and drugs for neuropathic pain, dementia or insomnia scored the highest in both dimensions.

In addition, we analyzed the correlation of potential advertisement spend among search engines, the correlation of volume among search engines, the correlation of Google’s *competition factor* and suggested bid, and the correlation of Google’s volume and spend (Fig 2E–2H). The correlation was high for all of them, except for competition against bid, as the *competition factor* depends on the number of advertisers for each query. The spend (volume * bid) correlated in both search engines (Fig 2E), the competition between advertisers and bid showed a lower correlation for Google (Fig 2F), reflecting that the competition is for the most expensive drugs. Average searches were highly correlated between search engines, with some exceptions for Google due to its dominant position (Fig 2G). As expected, correlation between spend and volume of searches was high for Google, with some exceptions where the volume was significantly higher than the spend (dots above the correlation line, Fig 2H), because not all diseases or drugs have the same interest for industry.

## Discussion

In this study, we evaluated the interest of the US population in neurological diseases by analyzing the frequency of web searches in the two most popular search engines. We found that the most common diseases were either very common diseases such as ADHD or dementia as well as medium frequency diseases with high social impact such as PD, MS or ALS. Regarding drugs prescribed in neurological diseases (including pain), the most commonly searched drugs were generic drugs (OTC) such as painkillers, or drugs for sleep disorders, dementia, ADHD, stroke or PD. Therefore, web searches provide an overview of the interests of the general population regarding neurological diseases. We must take in consideration that the results from web searches would not match results from epidemiological studies because they always involve the bias for the most educated population having better access to the Internet.

In addition, we evaluated the market value of such drugs for the healthcare industry, which is mainly based on direct advertisement to users and therefore differs from marketing strategies for addressing health care professionals such as neurologists (e.g. recently approved drugs for CNS diseases). This infodemiology analysis can be useful for defining the health policies for neurological diseases as well as understanding the interest of the pharmaceutical industry.

Previous studies regarding web searches about neurological diseases have shown that patients with several neurological diseases such as MS, Parkinson disease or epilepsy search extensively the web [6–14]. For example, in the case of status epilepticus, most people searched to obtain information on its definition, subtypes, and management and they came from both developed and underdeveloped countries [9]. Brigo and collaborators have shown that such searches were highly influenced by media releases related with celebrities, scientific discoveries or approval of new therapies [17, 18]. Although this may be a limitation of our study, we have surveyed that around the time of the study, no major media news related with brain diseases happened (e.g. a celebrity is diagnosed with such condition, launch of a new drug or an epidemic), although patient’s associations are constantly launching press released that altogether will buffer some of such effects.

Several limitations should be considered for the interpretation of this data. First, while these web searches cover the US population, they cannot be considered representative of the overall population interests because of differential access to the internet and computer literacy between ethnicities, ages and socio-economic status. We decided to focus only in the US in order to avoid other sources of bias. The US population is the most populated country of all developed countries plus they use a single language (although immigrants may use their local languages in their searches) with a common health system. We have considered analyzing other populous developed countries together (European Union and the Far East—Japan and South Korea-), but the language and the health systems diversity would decrease the accuracy of the results and would not solve the bias versus wealthy and well educated populations having better access to Internet. Moreover, we discarded underdeveloped countries in our study because they would introduce the bias of having different frequencies of diseases (e.g. infectious, nutrition, traumatic) and less access to therapies. In summary, our US study can be considered a good proxy to the frequency of neurological diseases and use of CNS drugs in developed countries and a baseline for future studies in other countries.

Second, search strategies can differ between people due to use of abbreviations, synonyms or colloquial expressions and here we have only analyzed disease terms and their abbreviations, but not other search strategies. We have explored the use of less specific terms in web searches such as symptoms and common complaints, but the specificity of such searches was low and the fact that the search engines do not allow to obtain user sessions, this prevents us to develop a strategy of linking concepts in order to increase sensitivity. That said, for drugs we used both the generic and brand names and therefore any variability due to polysemy or semantic strategies is less relevant. Our results may be influenced by media and news releases (e.g. celebrities being diagnosed, drug approvals, scientific discoveries or mass-media events), which can significantly drive the searches[17, 18]. Finally, this study did not identify users (computer IP) at any time and we used aggregated results. For this reason, we did not use session data, preventing us from performing a serial analysis of the searches from a given person, from the disease to the therapy or other related concerns. Although our analysis was restricted to a single year, this analysis can be performed on a yearly basis for future studies in order to reveal trends in the frequency or interest in neurological diseases.

## Supporting information

S1 File List of search terms. List of search terms used in this study.
(XLS)

S2 File Results searches neurological diseases. Results for brain disease searches.
(XLS)

S3 File Results searches CNS drugs. Results for CNS drug searches.
(XLS)

## Abbreviations

ADHD: Dementia or Attention Deficit/Hyperactivity Disorder
ALS: Amyotrophic Lateral Sclerosis
APIs: public advertising interfaces (APIs)
CNS: Central Nervous System
FDA: Federal Drug Administration
IBM: Inflammatory Body Myositis vs IBM corporation
MS: Multiple Sclerosis
PD: Parkinson disease
PSP: Progressive Supranuclear Palsy vs PlayStation Portable
TM: Transverse Myelitis vs Trademark
